# Higher phage virulence accelerates the evolution of host resistance

**DOI:** 10.1098/rspb.2022.1070

**Published:** 2022-10-12

**Authors:** Carolin C. Wendling, Janina Lange, Heiko Liesegang, Michael Sieber, Anja Poehlein, Boyke Bunk, Jelena Rajkov, Henry Goehlich, Olivia Roth, Michael A. Brockhurst

**Affiliations:** ^1^ GEOMAR Helmholtz Centre for Ocean Research Kiel, Marine Evolutionary Ecology, Düsternbrooker Weg 20, 24105 Kiel, Germany; ^2^ ETH Zürich, Institute of Integrative Biology, Universitätstrasse 16, CHN D 33, 8092 Zürich, Switzerland; ^3^ Department of genomic and applied microbiology, Georg-August-University Göttingen, Grisebachstr 8, 37077 Göttingen, Germany; ^4^ Max Planck Institute for Evolutionary Biology, August-Thienemann-Str. 2, 24306 Plön, Germany; ^5^ Department Bioinformatics and Databases, Leibniz Institute DSMZ-German Collection of Microorganisms and Cell Cultures, Inhoffenstr. 7B, 38114 Braunschweig, Germany; ^6^ Marine Evolutionary Biology, Kiel University, Am Botanischen Garten 1-9, 24118 Kiel, Germany; ^7^ Division of Evolution and Genomic Sciences, University of Manchester, Dover Street, Manchester M13 9PT, UK

**Keywords:** virulence, filamentous phages, experimental evolution, resistance evolution

## Abstract

Pathogens vary strikingly in their virulence and the selection they impose on their hosts. While the evolution of different virulence levels is well studied, the evolution of host resistance in response to different virulence levels is less understood and, at present, mainly based on observations and theoretical predictions with few experimental tests. Increased virulence can increase selection for host resistance evolution if the benefits of avoiding infection outweigh resistance costs. To test this, we experimentally evolved the bacterium *Vibrio alginolyticus* in the presence of two variants of a filamentous phage that differ in their virulence. The bacterial host exhibited two alternative defence strategies: (1) super infection exclusion (SIE), whereby phage-infected cells were immune to subsequent infection at the cost of reduced growth, and (2) surface receptor mutations (SRM), providing resistance to infection by preventing phage attachment. While SIE emerged rapidly against both phages, SRM evolved faster against the high- than the low-virulence phage. Using a mathematical model of our system, we show that increasing virulence strengthens selection for SRM owing to the higher costs of infection suffered by SIE immune hosts. Thus, by accelerating the evolution of host resistance, more virulent phages caused shorter epidemics.

## 1. Background

Infectious organisms vary strikingly in their level of virulence and the resulting selection they impose on hosts. Indeed, even closely related viruses, such as different strains of myxoma [1] or coronaviruses [2], can differ greatly in virulence. While the evolution of virulence has been studied extensively during the past two decades, both using selection experiments [3–5] and observations of parasites evolved in nature [6,7], how hosts respond to virulence-mediated selection is less well-explored. Our understanding of how virulence will impact evolutionary trajectories of resistance in a host population, and how these trajectories change with different levels of virulence, is mainly based on observational patterns [8,9] and theory [10–12], with few experimental tests [13]. In general, increased virulence strengthens selection for the evolution of host resistance if the costs of resistance are outweighed by the benefits of avoiding infection [10–12]. As such, at very low virulence, although infection is common, resistance is not favoured because the cost of resistance is likely to exceed any benefits of avoiding mild disease [11]. With increasing virulence, resistance is more strongly selected as the cost of resistance becomes outweighed by the detrimental effects of more severe disease, leading to the more rapid evolution of resistance [12]. However, at extreme levels of high virulence, selection for resistance can weaken once more. This is because high virulence reduces disease prevalence, weakening selection for costly resistance [11]. Experimental tests of these predictions are, however, rare.

To explore how different levels of virulence influence the dynamics of host resistance evolution, we designed a selection experiment using the model bacterium *Vibrio alginolyticus* K01M1 as a host and two variants of the filamentous phage, VALGΦ8, which differ in their virulence but are otherwise isogenic [14] (a detailed description of the two phage variants is given in §2 and tables 1 and 2). Filamentous phages (family *Inoviridae*)—i.e. long, thin proteinaceous filaments that contain a circular single-stranded DNA genome—are ideal model systems to study virulence evolution [3,5]. Filamentous phages establish chronic infections whereby virions are continuously released without lysis. Although filamentous phages do not kill their host, infections often result in reduced host growth rates [15,16]. This reduction in growth (and potentially even cell death) can result from overexpression of phage-encoded proteins inserted into the bacterial membrane, such as pI encoding a protein component of the transenvelope-assembly secretion system [15]. Thus, we define virulence as the reduction in bacterial growth resulting from phage infection. Accordingly, we quantify virulence directly by measuring the reduction in bacterial growth rate caused by phage infection relative to the growth rate of phage-free cultures.

**Table 1.** Strains (including NCBI accession numbers for chromosomes 1 and 2) used in the present study.

isolate	accession number(s)	genomic phages	role in evolution experiment
*V. alginolyticus* K01M1	CP017889.1	*Vibrio* phage VALGΦ6	host strain during evolution experiment
	CP017890.1		
*V. alginolyticus* K04M1	CP017891.1	*Vibrio* phage VALGΦ6	donor of the episomal, low-virulence phage VALGΦ8
	CP017892.1	*Vibrio* phage VALGΦ8	
*V. alginolyticus* K04M5	CP017899.1	*Vibrio* phage VALGΦ6	donor of the integrative, high-virulence phage VALGΦ8
	CP017900.1	*Vibrio* phage VALGΦ8

**Table 2.** Parameter values of mathematical model and their biological meaning.

parameter	biological meaning	value
*r*	maximum growth rate (mgr) of ancestor *B*	2.5 (h^−1^)
*K*	carrying capacity of bacteria	10^9^ cells ml^−1^
*w*	curvature parameter	0.02
*v*	virulence	variable
Φ	phage adsorption rate	10^−8^ (h^−1^)
*ß*	phage production rate	50 (phages cell^−1^ h^−1^)

During chronic infections, most phage genes are repressed to ensure host cell viability [17]. This is achieved through the action of prophage-encoded repressor proteins that also prevent superinfection, i.e. superinfection exclusion (SIE) by the same or closely related [18] phage(s). Many filamentous phages, including vibriophages from the present study, can provide SIE immunity by producing the phage-encoded receptor-binding protein pIII, which blocks primary and secondary phage receptors [15]. As such, chronically infected host cells become protected from subsequent infection through SIE. Alternatively, bacteria can acquire resistance to filamentous phage infection through mutations causing alterations to the surface receptors to which the phages bind, thus preventing phage infection [19]. How phage virulence alters selection for SIE versus surface receptor modification (SRM) resistance is unclear.

Combining experimental evolution with whole genome sequencing, we show that SIE immunity arose rapidly and at a similar rate against both phages. By contrast, SRM evolved more rapidly against the high- compared to the low-virulence phage, driving faster extinction of the high-virulence phage. Using an experimentally parameterized mathematical model, we show that accelerated replacement of SIE immunity by SRM was driven by increased infection costs, in terms of reduced growth, suffered by SIE immune hosts with increasing phage virulence. Resistance mutations were identified in genes encoding the mannose-sensitive hemagglutinin (MSHA) type IV pilus, which pleiotropically caused reduced motility of these resistant bacteria. Together these data show that higher phage virulence accelerated the evolution of resistance, consequently driving faster phage extinctions and shorter epidemics.

## 2. Material and methods

Experiments were conducted using the *Vibrio alginolyticus* strain K01M1 [20]. K01M1 contains one integrated filamentous *Vibrio* phage VALGΦ6 (later called: resident K01M1Φ-phage throughout the manuscript), replicating at a low frequency [14]. We compared VALGΦ6 and VALGΦ8 during previous work and found that both phages share relatively little sequence similarity (50.72%), except for proteins involved in DNA replication [14]. In a previous study we also confirmed that VALGΦ6 does not confer SIE to VALGΦ8 [21]. Compared to other, closely related *V. alginolyticus* strains, K01M1 is highly susceptible to infections by filamentous phages, including VALGΦ8 [21]. For the selection experiment, we used two different isogenic versions of the filamentous *Vibrio* phage VALGΦ8: VALGΦ8_K04M1_ (lower virulence) and VALGΦ8_K04M5_ (higher virulence; table 1), which have been isolated from two different hosts (*V. alginolyticus* K04M1 and *V. alginolyticus* K04M5). The main difference between these two VALGΦ8 variants lies within the intergenic region upstream of gene K04M5_41300, whose function and impact we cannot explain (electronic supplementary material, figure S1). While both phages have been shown to significantly reduce the growth of K01M1 [21,22], infections with the higher-virulence VALGΦ8_K04M5_ impose a significantly stronger reduction in bacterial growth (RBG) than infections with the low-virulence phage VALGΦ8_K04M1_ (RBG VALGΦ8_K04M5_ = 0.73_,_ RBG VALGΦ8_K04M1_ = 0.58; Welch two sample *t*-test: *t*_10.79_ = 16.406, *p* < 0.001). All experiments were carried out in liquid medium (medium101: 0.5% (w/v) peptone, 0.3% (w/v) meat extract, 3.0% (w/v) NaCl in MilliQ water) at 25°C in 30 ml microcosms containing 6 ml of medium with constant shaking at 180 rpm.

### (a) Selection experiment

Six replicate populations were founded for each of three treatments from independent clones of K01M1. Treatments comprised (a) the higher-virulence VALGΦ8_K04M1_, (b) the lower-virulence VALGΦ8_K04M5_ or (c) no phage as control. Each population was established from 60 µl of an independent overnight culture (5 × 10^8^ CFU ml^−1^). At the beginning of the experiment, we inoculated phage-containing treatments with 300 µl of a 5 × 10^10^ plaque-forming units (PFU) ml^−1^ stock solution. Populations were propagated by transferring 0.1% to fresh medium every 24 h for a total of 30 transfers. On transfer T0, T1 and T2, followed by every other transfer, phage and bacterial densities were determined, as described below, and whole population samples were frozen at −80° C at a final concentration of 33% glycerol. In addition, on transfers T0, T1, T2 and T6, followed by every sixth transfer, 24 single colonies were isolated randomly from each population and stored at −80° C. These colonies were later used during follow-up assays, as described below. Two populations from the control treatment that tested positive for phage infection, indicating contamination, were excluded from later assays.

### (b) Bacterial and phage densities

*Bacterial densities*: bacterial densities were determined by plating out 100 µl of a dilution series ranging from 10^−5^ to 10^−7^ on *Vibrio* selective Thiosulfate Citrate Bile Sucrose Agar (TCBS) plates (Sigma Aldrich). Plates were incubated overnight at 25°C, and the total numbers of colonies were counted the following day.

*Phage densities*: quantification of filamentous phages by standard spot assays is often impossible [23]. Instead of typical lytic plaques, we mostly observed opaque zones of reduced growth. Thus, we used spectrometry to quantify phage prevalence (http://www.abdesignlabs.com/technical-resources/bacteriophage-spectrophotometry), which uses the constant relationship between the length of viral DNA and the amount of the major coat protein VIII of filamentous phages, which, together, are the main contributors to the absorption spectrum in the UV range. The number of phage particles per ml can be calculated according to the following formula:

$$\frac{\mathrm{phages}}{\mathrm{ml}}=\frac{(\mathrm{OD}269-\mathrm{OD}320)*6e16}{\mathrm{bp}},$$

where OD269 and OD320 stand for optical density at 269 and 320 nm, respectively, and bp stands for a number of base pairs per phage genome.

The method is based on small-scale precipitation of phages by single PEG precipitation. After centrifuging 1500 µl of the phage containing overnight culture at 13 000×g for 2 min, 1200 µl of the supernatant were mixed with 300 µl PEG/NaCl 5× and incubated on ice for 30 min. Afterwards, phage particles were pelleted by two rounds of centrifugation at 13 000×*g* for 2 min, resuspended in 120 µl TBS 1× and incubated on ice. After 1 h, the suspension was cleaned by centrifugation at 13 000×g for 1 min, and absorbance was measured at 269 and 320 nm.

Quantifying filamentous phages using spectrometry is likely erroneous if the viral load is low. Therefore, we additionally quantified phage prevalence/phage extinction in each of the populations on every second transfer day by standard spot assays. To do so, we used a serial dilution (up to 10^−6^) of the phage-containing supernatant on the ancestral host (for details, see [21]) and recorded the dilution at which the typical opaque zones of reduced bacterial growth became visible.

### (c) Measuring phage-defence

We quantified resistance to the respective ancestral phage by determining the reduction in bacterial growth rate (RBG) imposed by the phage, adapted from [24] with some modifications according to [25]. Twenty-four random colonies from each population from transfers T0, T1, T2, T6, T12, T18, T24 and T30 were introduced into 96-well microtiter plates containing Medium101 at a concentration of 5 × 10^6^ cells ml^−1^ and inoculated with approximately 2.5 × 10^6^ PFU ml^-1^ of the respective ancestral phage used for the selection experiment or without phage (control). Absorbance at 600 nm was measured using an automated plate reader (TECAN infinite M200) at T0 and again after 20 h of static incubation at 25° C. The reduction in bacterial absorbance ‘RBG’ was calculated according to the following formula:

$$\mathrm{RBG}=\frac{\mathrm{OD}600(t=20)-\mathrm{OD}600(t=0)[\mathrm{Phage}]}{\mathrm{OD}600(t=20)-\mathrm{OD}600(t=0)[\mathrm{Control}]},$$

where OD stands for optical density at 600 nm.

### (d) Frequency of prophage carriage

On transfer T0, T1, T2, T6 followed by every sixth transfer, we measured the frequency of phage carriage of 24 random clones per population using standard PCR. We designed primers (VALGΦ8_Forward TGGAAGTGCCAAGGTTTGGT, VALGΦ8_Revers GAAGACCAGGTGGCGGTAAA) that specifically target the *Vibrio* phage VALGΦ8, but not the ancestral VALGΦ6, using the NCBI Primer-BLAST webpage (http://www.ncbi.nlm.nih.gov/tools/primer-blast/). Note, these primers only detect the presence of VALGΦ8, but not whether it exists episomally or as a prophage integrated into the chromosome. Glycerol stocks were inoculated overnight (25° C, 180 rpm) in Medium101 and subsequently diluted (1 : 10) in HPLC purified H_2_O and frozen at −80° C. One µl of this suspension was used as DNA template in the PCR assay. Reaction comprised 1 µl Dream Tag Buffer, 0.1 µl Dream Tag DNA polymerase (Thermo Scientific, USA), 4.9 µl H_2_O, 1 µl dNTPs [5 mM] and 1 µl of each primer [50 µM]. The amplification program used consisted of: (i) 3 min at 95° C, (ii) 35 cycles of 45 sec at 95° C, 30 sec at 63° C, 45 sec at 72° C, (iii) 7 min at 72° C. Afterwards, 5 µl of each reaction were mixed with 2 µl loading dye (10×) and loaded onto a 1.2% agarose gel dissolved in 1×TAE gel buffer. GeneRuler Express DNA-ladder was used as a size marker. Agarose gels were run for 15 min at 70 V in 0.5× TAE running buffer and subsequently stained with ethidium bromide for 10 min. DNA was visualized using UV light, and documentation took place using Intas Gel iX20 Imager. Phage presence was recorded as positive if a PCR product of 1209 bp was visible.

For all subsequent assays, we randomly picked one resistant clone with a positive PCR product (later called: super infection exclusion SIE hosts) and one resistant clone with a negative PCR product (later called: surface receptor mutant (SRM)) from each phage-evolved population as well as two randomly selected non-resistant clones from the control populations.

### (e) Competition experiments

To determine differences in fitness between the two resistance forms, we measured the competitive fitness of three randomly selected PCR-positive relative to three randomly selected PCR-negative clones from each treatment. Each competition culture was done in triplicates as described in [26]. In brief, overnight cultures of both competing strains (one labelled with a green fluorescent protein (GFP)-marker) were mixed 1 : 1, and 60 µl of this mixture were inoculated to 6 ml Medium 101 to initiate each competitive culture. After 24 h, fitness was estimated by flow cytometry (FACS-Caliburm Becton & Dickinson, Heidelberg, Germany), where absolute fluorescent and non-fluorescent cells were calculated. Competitive fitness was estimated as the ratio in Malthusian parameters [27]:

$$W=\frac{\ln{\left({\mathrm{abundance}}_{t=24}/{\mathrm{abundance}}_{t=0}\right)}_{\mathrm{competitor}1}}{\ln{\left({\mathrm{abundance}}_{t=24}/{\mathrm{abundance}}_{t=0}\right)}_{\mathrm{competitor}2}}.$$

### (f) Bacterial growth rate and phage production

To determine fitness parameters that could explain observed differences in competitive fitness, we additionally quantified bacterial growth rate (*µ*) by means of 24h growth curves and phage production using PEG precipitation (as described in §2b) of the same clones used for the competition assays (i.e., one SIE and one SRM host from each phage-treated population and two random phage-susceptible clones from the control populations plus ancestors).

### (g) Motility

Motility was visualized on mid-exponential growth cultures using a light microscope, and swimming was captured for 50 s.

### (h) Whole genome sequencing

We used a combination of long- and short-read sequencing to obtain complete genomes of the same clones from the assays above. To do so, we picked one SIE and one SRM host from each phage-treated population and one random phage-susceptible clone from each control population. Clones were taken from timepoint 2 because phage carriers disappeared quickly from the populations, and we were thus unable to pick one mutant and one SIE host from the same timepoint and population later than timepoint two. A full description of sequencing and genomic analysis is given in the electronic supplementaryl material.

### (i) Statistical analysis

All statistics were performed in the R 4.0.4 statistical environment, R core Team (2020). For all analysis that aimed to compare the two different phage treatments with one another, control populations (i.e., those that evolved without phages) were excluded. When comparing temporal dynamics between phage treatments, we excluded the starting timepoint T0 because these measurements were taken before phages were added to the populations. Full description of the statistical analysis is given in the electronic supplementaryl material.

### (j) Mathematical model

We modelled the dynamics of the non-resistant evolved clones (with density *B*), resistant SIE hosts (*I*), resistant SRM hosts (*R*) and SIE hosts that have also acquired the MSHA mutation (*IR*), as well as the phage population (*V*) in batch cultures using a system of differential equations. A full description of the modelling section is given in the electronic supplementaryl material.

## 3. Results

### (a) Initial ecological dynamics vary according to phage virulence

**Figure 1.**
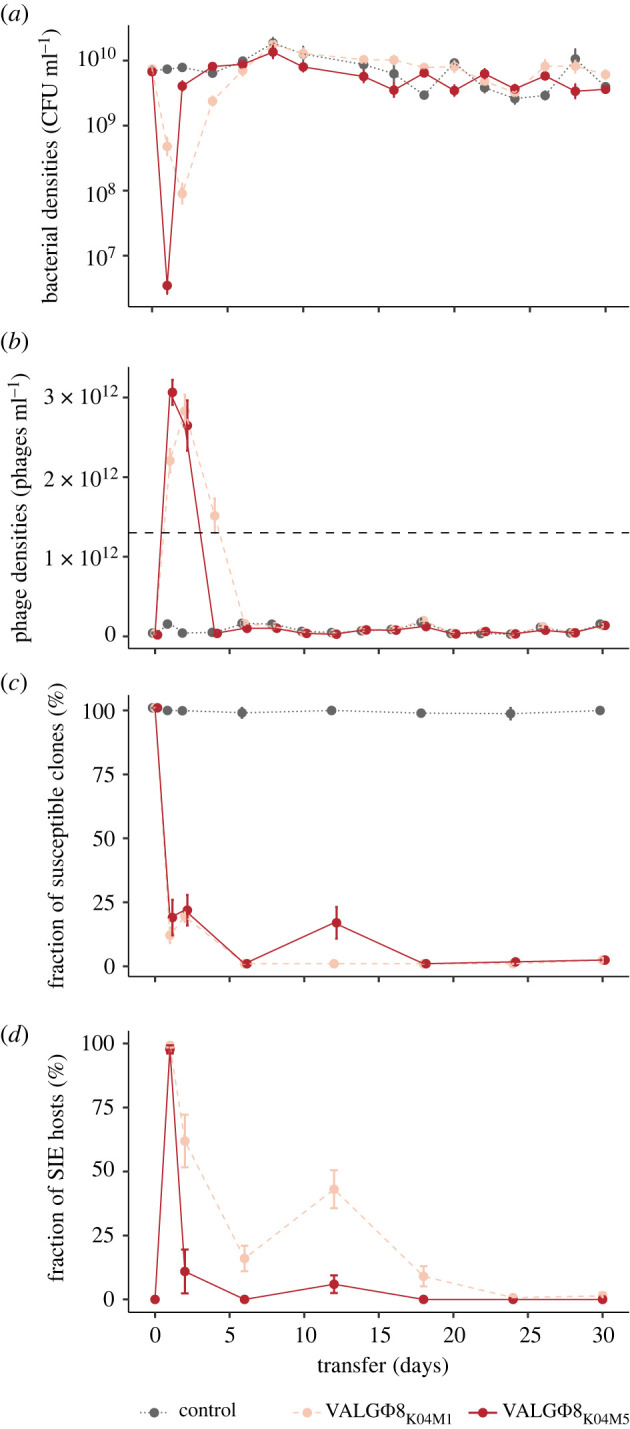
Population dynamics over 30 transfers. (*a*) Bacteria in CFU ml^–1^. (*b*) Phages in PFU ml^–1^; the horizontal grey dashed line represents the quantification limit below which quantifying filamentous phages using spectrophotometry is inaccurat. Note: free phages in the control treatment stem from the low-replicating resident phage VALGΦ6 (table 1). (*c*) Fraction of susceptible clones (%; *n* = 24). (*d*) Fraction of SIE hosts within phage-resistant clones. Fractions are based on 24 random clones per replicate population per timepoint. In all panels, data are represented as means of six replicate populations per treatment; error bars represent standard errors. Colours correspond to one of three experimental treatments: lower-virulence VALGΦ8_K04M1_ (light red, dashed), higher-virulence VALGΦ8_K04M5_ (dark red) and no phage (grey, dashed-dotted). (Online version in colour.)

**Figure 2.**
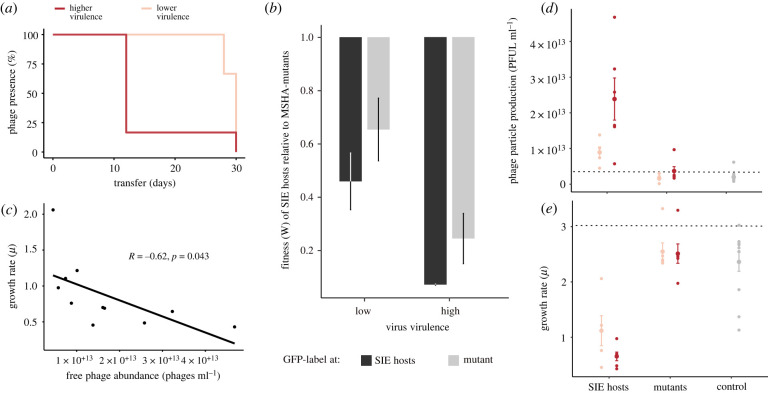
Phage prevalence (*a*) and fitness effects of evolved phage resistance versus immunity (*b–e*): (*a*) Phage prevalence for each co-evolving population in the presence of higher-virulence phage VALGΦ8_K04M5_ (dark red) or the lower-virulence phage VALGΦ8_K04M1_ (light red) over 30 transfers. (*b*) Darwinian fitness of SIE relative to SRM hosts. A value of unity corresponds to equal fitness. To account for potential costs associated with the GFP protein, competitions were performed where either the SIE or the SRM host was labelled (*n* = 3). (*c*) Correlation between bacterial growth rate [*µ*] and production of free phages measured as PFU ml^–1^ per clone. (*d*) Phage particle production [PFU ml^–1^] and (*e*) growth rate *µ*: both measured after 24 h of bacterial growth for SIE hosts, SRM hosts, clones from the control populations (grey) and the ancestral K01M1 strain (dotted horizontal line). Clones exposed to lower virulent VALGΦ8_K04M1_ are shown in light red, clones exposed to higher virulent VALGΦ8_K04M5_ in dark red. Phages from the ancestral K01M1, from SRM hosts and the control clones stem from an ancestral filamentous *Vibrio* phage VALGΦ6 integrated on chromosome 2 of K01M1 (table 1). Shown are means ± s.e., *n* = 24. (Online version in colour.)

To explore how variation in virulence influences the dynamics of host resistance evolution, we experimentally evolved the bacterium *Vibrio alginolyticus* K01M1 with or without one of two isogenic filamentous phages that differ in their virulence for 30 serial transfers (approx. 240 bacterial generations). The high virulence phage VALGΦ8_K04M5_ reduces bacterial growth by 73%, whereas the low-virulence phage VALGΦ8_K04M1_ reduces bacterial growth by 58% (table 1). We first compared the ecological dynamics of bacterial and phage populations between treatments. Phages reduced bacterial densities by several orders of magnitude in both phage treatments compared to no phage control populations (figure 1*a*). The immediate reduction (measured 24 h post infection [hpi]) in bacterial density was greater in populations exposed to the higher virulence phage (VALGΦ8_K04M5_) than the lower-virulence phage (VALGΦ8_K04M1_; figure 1*a*). Correspondingly, in both treatments, phages amplified massively and rapidly, reaching 3.01 × 10^12^ PFU/ml (VALGΦ8_K04M5_) 24 hpi and 2.83 × 10^12^ PFU ml^−1^ (VALGΦ8_K04M1_) 48 hpi (figure 1*b*), before declining to levels comparable to control populations (note that the genome of *V. alginolyticus* K01M1 contains a resident phage, VALGΦ6, that produces phage particles at a low background rate). These data suggest that the strong reduction in bacterial densities at the beginning of the experiment (figure 1*a*) directly resulted from the costly production of viral particles (figure 1*b*). Over time, however, the densities of bacterial populations exposed to the higher virulence phage recovered three times faster than populations exposed to the lower-virulence phage (significant phage:transfer interaction in gls-model: *F*_15,186_ = 6.58, *p* < 0.001, figure 1*a*). Declining phage densities in both treatments accompanied bacterial population recovery. Still, phage survival varied according to phage virulence (log-rank test: Chisq_1_ = 4.9, *p* = 0.03), with the higher-virulence phage going extinct more rapidly than the lower-virulence phage (figure 2*a*).

### (b) Rapid emergence of superinfection exclusion immunity

These bacteria–phage population dynamics suggest that the emergence of bacterial defences against phage infection may have enabled the recovery of the host population. Consistent with this hypothesis, the proportion of susceptible hosts rapidly declined to zero within 24 h in both treatments and remained so for the duration of the experiment (figure 1*c*). Bacteria can develop protection from filamentous phage infection by two distinct mechanisms: superinfection exclusion (SIE) immunity, where already infected cells are protected from subsequent infection by the same phage through phage-encoded genes [28–31], or resistance, for instance via modification of the bacterial phage receptor, preventing phage from entering the host cell [19]. To quantify the frequency of SIE immunity, we used PCR with primers that specifically target VALGΦ8 to test for the presence of the relevant phage in the bacterial genome (the presence of a PCR product suggests SIE owing to the presence of VALGΦ8). SIE rapidly increased in frequency and dominated bacterial populations in both treatments after 24 h (figure 1*d*). However, after 48 h, the proportion of SIE hosts began to decline, and did so significantly faster in populations exposed to the higher-virulence phage (figure 1*d*, significant phage:transfer interaction in a general linear model: *F*_6,60_ = 10.18, *p* < 0.001). Given that these populations contained no susceptible bacteria from 24 h onwards (out of 24 tested colonies per timepoint), the subsequent decline of SIE hosts suggests their displacement by the invasion of resistant genotypes, and the higher-virulence phage was more strongly selected for this.

### (c) Resistance is associated with mutations in MSHA type IV pilus-encoding genes

**Figure 3.**
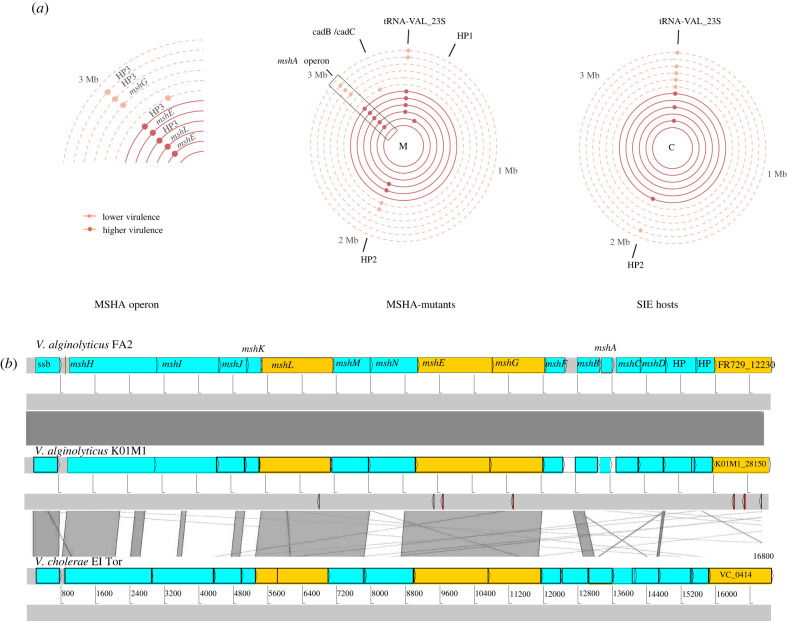
(*a*) Genetic loci on chromosome 1 under positive selection as indicated by parallel genomic evolution in populations exposed to phages: right: SIE hosts; middle: SRM hosts; left: zoom into MSHA-operon region from SRM hosts. Only loci that are not present in control populations are shown. Concentric circles correspond to one clone isolated from either the higher-virulence VALGΦ8_K04M5_ (six inner circles, dark red) or the lower-virulence VALGΦ8_K04M1_ phage (six outer circles, light red). Each small dot corresponds to one mutation event on the respective clone. HP, hypothetical protein; HP3 corresponds to locus tag K01M1_28150. For more detailed information on the underlying mutation, see electronic supplementary material, table S1. (*b*) Structure of the MSHA-operon and comparative genomics comprising MSHA-operons from *V. alginolyticus* FA2 (top), *V. alginolyticus* K01M1 and *V. cholerae* El Tor (bottom). Similarity between regions is indicated by dark grey blocks, genes with detected mutations are marked in orange, detected mutations are marked as arrows below *V. alginolyticus* K01M1. (Online version in colour.)

To test whether the decline of SIE hosts after 24 h was driven by the invasion of surface receptor modification (SRM) resistance, we used whole genome sequencing (WGS) of two randomly chosen clones from each population isolated at transfer 2. This included one PCR-positive clone (SIE) and one PCR-negative clone (resistant but not phage-carrying) to identify mutations. We observed no loci with mutations on chromosome 2 or the plasmid pl9064. However, on chromosome 1 we identified 12 loci with mutations that were not present in clones from the control treatment, suggesting that these were associated with phage-mediated selection. Of these 12 loci, two were affected in PCR-positive and PCR-negative clones. This included an intergenic region between tRNA-Val and the 23S ribosomal RNA that has been repeatedly hit in both clone types and phage treatments but whose function we cannot explain. The remaining ten loci were exclusive to PCR-negative clones, suggesting a potential role in evolved phage resistance. Out of these nine loci, eight had substitutions, duplications, insertions or deletions in four different genes belonging to the MSHA type IV pilus operon (*mshL, mshE, mshG, K01M1_28150*; figure 3*a* and electronic supplementary material, table S1). Among those, three caused severe frameshift mutations that presumably have a high impact on the function of these proteins. While the locus *K01M1_28150* was affected twice in both phage treatments, mutations in *mshL* and *mshE* occurred exclusively in response to the higher-virulence phage and mutations in *mshG* in response to the lower-virulence phage. Moreover, we found more mutated MSHA-loci among clones exposed to the higher-virulence (5/6) compared to the lower-virulence phage (3/6). This supports our previous findings, suggesting a stronger selection for resistance against the higher-virulence phage.

The absence of mutated MSHA-loci in PCR-positive clones and a high prevalence in PCR-negative clones (8/12) suggest a strong parallel evolution of phage resistance. The MSHA operon is highly conserved across *Vibrio* clades (figure 3*b*), and we found one corresponding orthologue to each gene in the *V. cholerae* El Tor MSHA operon (figure 3*b*). This suggests that, similar to other *Vibrios* [19], the MSHA type IV pilus plays an important role in resistance against VALGΦ8. Note that searching all assembled genomes for CRISPR-associated and CRISPR array-like repetitive sequence patterns did not yield any results. From here onwards, all PCR-negative phage-resistant clones are referred to as SRM hosts. The genomic data also confirmed that clones with a positive PCR result (i.e., SIE host) all contained the respective phage genome, which did not integrate into the chromosome but existed episomally in all sequenced clones (electronic supplementary material, table S2; figures S3).

We found four PCR-negative clones that were resistant to infections with ancestral phages but that did not acquire mutations within the MSHA operon. One explanation for this could be phenotypic resistance, where phage adsorption to bacteria is strongly reduced [32]. Another explanation could be inactivation of genes required for phage replication [33]. For instance, we found two PCR-negative clones with mutations in hypothetical proteins whose functions we do not know.

### (d) Virulence determines the rate of resistance evolution in a mathematical model

**Figure 4.**
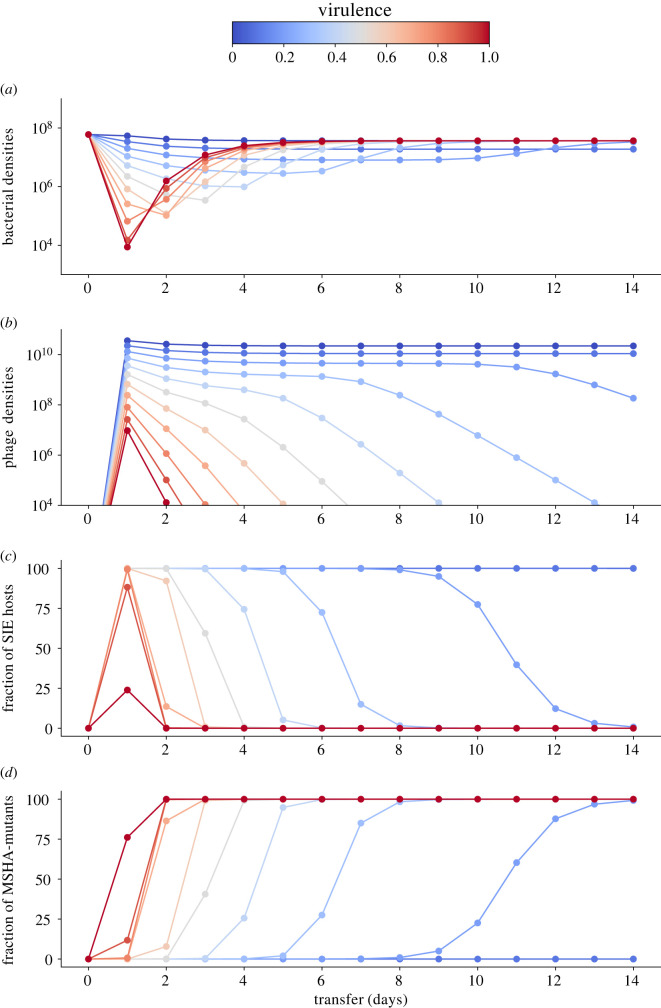
Results of model simulations of 14 transfers for (*a*) bacteria in CFU ml^–1^, (*b*) phages in PFU ml^–1^, (*c*) SIE hosts and (*d*) SRM hosts depending on phage virulence (colour coded from blue: no virulence to red: high virulence). (Online version in colour.)

To generalize our findings across a wider range of virulence levels we developed an experimentally parameterized mathematical model. As in the experiment, bacterial densities dropped by several orders of magnitude upon phage infection (figure 4*a*). By simulating the infection dynamics over a wider range of virulence levels, we found that this drop occurred later and was less strong with decreasing virulence. While phage densities, irrespective of virulence, peaked 24 hpi, phages persisted for longer and at higher levels when they were less virulent (figure 4*b*). Similar to the experiment, the model predicts that SIE immunity emerges rapidly within 24 hpi (figure 4*c*) but will only reach high levels if virulence is less than 1. To capture the displacement of SIE by SRM hosts, we implemented a cost of reduced growth for SIE hosts that is directly linked to virulence (figure 2*c*), i.e., the higher the virulence of the infecting phage, the lower the growth rate of the SIE host. SRM hosts grew at the same rate as the non-resistant clones (figure 2*e*).

We found that SRM hosts increased and replaced SIE hosts faster with increasing levels of virulence (figure 4*c,d*). Our model shows that this replacement occurs across a wide range of virulence levels, which we were not able to capture in the experiment. The faster replacement of SIE by SRM hosts at higher virulence levels is driven by higher costs, i.e., reduced growth of infection in SIE hosts, which increase monotonically with increasing phage virulence. Overall, our simulations predict that selection for resistance increases with virulence and is directly related to the costs of SIE. Thus, resistance is more likely to evolve against higher-virulence infections.

### (e) Relative fitness of surface receptor mutants increases with phage virulence

To directly test our model predictions that the fitness benefit of SRM relative to SIE immunity increases with increasing phage virulence, we performed a pairwise competition experiment in which we quantified the relative fitness of SRM against SIE hosts (figure 2*b*). The fitness benefit of the resistance mutation was higher against bacteria carrying the higher-virulence phage than bacteria carrying the lower-virulence phage (significant treatment term in a linear model with treatment, GFP-label and the interaction thereof as fixed factors: *F*_1,8_ = 18.63, *p* = 0.003, electronic supplementary material, table S3). These fitness data are consistent with the more rapid decline of VALGΦ8_K04M5_-carriers than VALGΦ8_K04M1_-carriers observed in the selection experiment and consistent with model predictions that suggest stronger selection for SRMs when exposed to a higher-virulence phage. This explains the dynamics of the SIE hosts in the selection experiment, which went to extinction in five out of six populations exposed to the higher-virulence phage 12 days post infection. However, when exposed to the lower-virulence phage, SIE hosts were able to persist until the end of the experiment, albeit at very low frequencies, in five out of six populations.

Bacterial population densities during the selection experiment were negatively correlated with the number of SIE hosts per population (Spearman correlation without zero inflation Φ-K04M1: *r* = −0.59, S = −3225.1, *p* = 0.002, Φ-K04M5: *r* = −0.7, S = 204.6, *p* = 0.04; electronic supplementary material, figure S4). This implies that, even though the majority of the clones in the populations were protected from further infection, bacterial populations were unable to recover as long as the dominating mechanism of defence was SIE immunity, presumably owing to virulence, resulting from the strong reduction in bacterial growth rate. To test this, we quantified differences in phage production and tested if phage production impaired bacterial growth in SIE hosts. VALGΦ8_K04M5_-carriers produced approximately 3.5 times more phage particles than VALGΦ8_K04M1_-carriers (VALGΦ8_K04M5_: mean = 2.39 × 10^13^ PFU ml^−1^ ± 1.44 × 10^13^, VALGΦ8_K04M1_: mean = 8.92 × 10^12^ PFU ml^−1^ ± 3.43 × 10^12^, figure 2*d*), and phage production significantly impaired bacterial growth (significant negative correlation between the amount of produced phages and bacterial growth rate, figure 2*c*). Direct comparisons of growth rates among resistant clones showed that SIE hosts also grew slower than SRM hosts (VALGΦ8_K04M5_: Wilcoxon rank sum test: *p* = 0.005; VALGΦ8_K04M1_: paired *t*-test: *t*_6.5_ = −4.58, *p* = 0.003, figure 2*e*). Together, these data suggest that SIE buys time, offering protection against subsequent infection, but at the cost of suffering the virulence of being infected. As in the model, where we predicted that the costs of SIE increase monotonically with phage virulence, SIE is eventually replaced by SRM, which happens faster with increasing levels of virulence, where the fitness benefits of SRM are greater. Ultimately dominance of host populations by SRM hosts resulted in faster extinction of higher-virulence phages, which were unable to overcome evolved host resistance.

### (f) Resistance leads to secondary costs

Lastly, we tested whether the observed mutations in the MSHA-pilus genes impair bacterial motility. We observed reduced swimming motility of SRM hosts compared to ancestral bacterial strains (electronic supplementary material, Video).

## 4. Discussion

Theory predicts that increasing virulence results in stronger selection for host resistance if the benefits of avoiding infection are higher than the costs of resistance [10–12], but experimental data are rare [13]. Using two filamentous phages that differ in their virulence in a selection experiment, we found that SIE immunity arose rapidly and at a similar rate irrespective of phage virulence. By contrast, SRM, which replaced SIE immunity, evolved more rapidly against the high- compared to the low-virulence phage. A limitation of our experimental system is that it contained only two virulence levels. Additional viral genotypes representing a wider range of virulence levels would be required to test the linearity (or nonlinearity) of the relationship between viral virulence and selection for host resistance. Thus, we developed a mathematical model of our system to generalize our findings across a broader range of virulence levels. The model confirmed that increasing virulence strengthens selection for SRM because SIE immunity becomes more costly with increasing virulence. Thus, higher levels of SRMs in the host population caused faster phage extinction and, ultimately, shorter epidemics.

Resistance arose from mutations in genes encoding the MSHA type IV pilus, a common receptor for filamentous phages [16]. These mutations reduced the motility of resistant clones, suggesting pleiotropic fitness costs of phage resistance mutations. Such secondary costs, i.e., reduced motility or even pathogenicity, are commonly observed during phage resistance evolution involving multifunctional structures on bacterial cell surfaces; for a review, see [34]. In many natural environments, motility is an important pathogenicity factor [35], which is often crucial for establishing acute infections but less important during chronic infections of eukaryotic hosts. A prominent example is cystic fibrosis (CF) lung infections by *Pseudomonas aeruginosa*. While initial colonizers are motile, adaptation to CF lungs during chronic infections is often characterized by loss of motility [36,37]. Thus, we predict that the replacement of SIE hosts by non-motile SRM hosts may be constrained to laboratory environments but may also occur in biofilms, where such antagonistic pleiotropic costs of surface receptor modifications are lower than the costs of SIE. By contrast, selective pressures occurring in eukaryotic hosts, such as resource competition, might reverse this effect and explain why filamentous phages, including highly virulent versions of VALGΦ8, persist in environmental isolates [14].

Filamentous phages are very common features of bacterial genomes [38], including those of environmental *Vibrio* strains closely related to our model strain K01M1, of which all carry VALGΦ6 and more than 50% VALGΦ8 [14]. While incorporating filamentous phage genomes into their own genome (but not necessarily into the bacterial chromosome) provides bacteria with immunity to future infection—through SIE immunity mediated by phage-encoded genes—we show that this comes at a fitness cost that scales positively with the virulence of the phage. Higher phage virulence selects for a faster replacement of SIE immunity with SRM, causing phage extinction (figure 2*a*). Thus, our data suggest that to be able to establish long-term chronic infections, filamentous phages have two options: they could evolve very low levels of virulence [39], such that the resulting cost of virulence is outweighed by the cost of resistance mutations. Alternatively, they could contribute positively to bacterial fitness by providing beneficial ecological functions [16]. Those benefits may derive either from phage-encoded gene functions, e.g., toxins [40–42], or from properties of the phage particles themselves, e.g., forming the biofilm extracellular-matrix [43], or acting as decoys for the vertebrate immune response [44]. Phage-mediated fitness benefits are often environmentally dependent [41,45–47], and the prevalence of filamentous phages in bacterial genomes is higher in those isolated from eukaryotic infections—where filamentous phages often encode important enterotoxins—than in those isolated from natural environments [15]. Even though we have not yet identified any associated ecological benefits, the high prevalence of VALGΦ8 in natural *V. alginolyticus* isolates [14] suggests that this phage may provide a selective advantage outside the laboratory in its natural environment, i.e., the pipefish. Conversely, however, bacterial genomes are graveyards of defective prophages [48], including filamentous phages [49], suggesting that decay, rather than peaceful coexistence, maybe a common outcome for phages integrated into bacterial genomes. Ultimately, their level of virulence will dictate the fate of filamentous phages: lower-virulence variants may enter into stable coexistence, while higher-virulence variants will be more prone to resistance-driven extinction and mutational decay if they do not provide a selective advantage.

## 5. Conclusion

Using a combination of experimental evolution, genomics and mathematical modelling, we show that higher phage virulence more strongly selects for resistance mutations in bacterial populations. With increasing phage virulence, resistance more rapidly displaces SIE from the bacterial host population, because SIE becomes progressively costlier. By accelerating the evolution of resistance, higher virulence drives phage extinctions, thus shortening viral epidemics. Our findings help to explain the extensive variation in virulence levels that exists among genotypes for a wide range of viruses. Moreover, our findings predict that to persist in the long-term, filamentous phages must either evolve low virulence or encode traits that are beneficial for their bacterial hosts.

## Data Availability

All experimental data have been deposited in the Dryad Digital Repository: doi:10.5061/dryad.kprr4xh6c [50]. Genomic data are available at NCBI: BioProject PRJNA735604, and in the supplemental data file electronic supplementary material, table S1 and S2. Supplemental information is provided in electronic supplementary material [51].

## References

[1] Fenner F, Marshall ID. 1957 A comparison of the virulence for European rabbits (*Oryctolagus cuniculus*) of strains of myxoma virus recovered in the field in Australia, Europe and America. J. Hyg. **55**, 149-191. (10.1017/S0022172400037098) 13439170 PMC2217926

[2] Weiss SR, Leibowitz JL. 2011 Chapter 4 - Coronavirus Pathogenesis. Adv. Virus Res. **81**, 85-164. (10.1016/B978-0-12-385885-6.00009-2) 22094080 PMC7149603

[3] Messenger SL, Molineux IJ, Bull JJ. 1999 Virulence evolution in a virus obeys a trade-off. Proc. R. Soc. B **266**, 397-404. (10.1098/rspb.1999.0651) PMC1689683 10097397

[4] Turner PE, Cooper VS, Lenski RE. 1998 Tradeoff between horizontal and vertical modes of transmission in bacterial plasmids. Evolution **52**, 315-329. (10.1111/j.1558-5646.1998.tb01634.x) 28568337

[5] Bull JJ, Molineux IJ, Rice WR. 1991 Selection of benevolence in a host–parasite system. Evolution **45**, 875-882. (10.1111/j.1558-5646.1991.tb04356.x) 28564051

[6] Herre EA. 1993 Population structure and the evolution of virulence in nematode parasites of fig wasps. Science **259**, 1442-1445. (10.1126/science.259.5100.1442) 17801279

[7] Ebert D. 1994 Virulence and local adaptation of a horizontally transmitted parasite. Science **265**, 1084-1086. (10.1126/science.265.5175.1084) 17832903

[8] Gates DE, Staley M, Tardy L, Giraudeau M, Hill GE, McGraw KJ, Bonneaud C. 2021 Levels of pathogen virulence and host resistance both shape the antibody response to an emerging bacterial disease. Sci. Rep. **11**, 8209. (10.1038/s41598-021-87464-9) 33859241 PMC8050079

[9] Kraaijeveld AR, Godfray HCJ. 1999 Geographic patterns in the evolution of resistance and virulence in *Drosophila* and its parasitoids. Am. Nat. **153**, S61-S74. (10.1086/303212) 29578778

[10] Boots M, Haraguchi Y. 1999 The evolution of costly resistance in host-parasite systems. Am. Nat. **153**, 359-370. (10.1086/303181) 29586625

[11] Restif O, Koella JC. 2003 Shared control of epidemiological traits in a coevolutionary model of host-parasite interactions. Am. Nat. **161**, 827-836. (10.1086/375171) 12858269

[12] van Baalen M. 1998 Coevolution of recovery ability and virulence. Proc. R. Soc. B **265**, 317-325. (10.1098/rspb.1998.0298) PMC1688890 9523434

[13] Kraaijeveld AR, Godfray HC. 1997 Trade-off between parasitoid resistance and larval competitive ability in *Drosophila melanogaster*. Nature **389**, 278-280. (10.1038/38483) 9305840

[14] Chibani CM, Hertel R, Hoppert M, Liesegang H, Wendling CC. 2020 Closely related *Vibrio alginolyticus* strains encode an identical repertoire of caudovirales-like regions and filamentous phages. Viruses **12**, 1359. (10.3390/v12121359) 33261037 PMC7761403

[15] Mai-Prochnow A, Hui JG, Kjelleberg S, Rakonjac J, McDougald D, Rice SA. 2015 Big things in small packages: the genetics of filamentous phage and effects on fitness of their host. FEMS Microbiol. Rev. **39**, 465-487. (10.1093/femsre/fuu007) 25670735

[16] Hay ID, Lithgow T. 2019 Filamentous phages: masters of a microbial sharing economy. EMBO Rep. **20**, e47427. (10.15252/embr.201847427) 30952693 PMC6549030

[17] Bondy-Denomy J, Davidson AR. 2014 When a virus is not a parasite: the beneficial effects of prophages on bacterial fitness. J. Microbiol. **52**, 235-242. (10.1007/s12275-014-4083-3) 24585054

[18] Refardt D. 2011 Within-host competition determines reproductive success of temperate bacteriophages. ISME J. **5**, 1451-1460. (10.1038/ismej.2011.30) 21412345 PMC3160688

[19] Jouravleva EA, McDonald GA, Marsh JW, Taylor RK, Boesman-Finkelstein M, Finkelstein RA. 1998 The *Vibrio cholerae* mannose-sensitive hemagglutinin is the receptor for a filamentous bacteriophage from *V. cholerae* O139. Infect. Immun. **66**, 2535-2539. (10.1128/IAI.66.6.2535-2539.1998) 9596713 PMC108235

[20] Chibani CM, Roth O, Liesegang H, Wendling CC. 2020 Genomic variation among closely related *Vibrio alginolyticus* strains is located on mobile genetic elements. BMC Genomics **21**, 354. (10.1186/s12864-020-6735-5) 32393168 PMC7216594

[21] Wendling CC, Piecyk A, Refardt D, Chibani C, Hertel R, Liesegang H, Bunk B, Overmann J, Roth O. 2017 Tripartite species interaction: eukaryotic hosts suffer more from phage susceptible than from phage resistant bacteria. BMC Evol. Biol. **17**, 1-12. (10.1186/s12862-017-0930-2) 28399796 PMC5387238

[22] Wendling CC, Goehlich H, Roth O. 2018 The structure of temperate phage–bacteria infection networks changes with the phylogenetic distance of the host bacteria. Biol. Lett. **14**, 20180320. (10.1098/rsbl.2018.0320) 30429242 PMC6283929

[23] Rakonjac J, Bennett NJ, Spagnuolo J, Gagic D, Russel M. 2011 Filamentous bacteriophage: biology, phage display and nanotechnology applications. Curr. Issues Mol. Biol. **13**, 51-76. (10.21775/cimb.013.051) 21502666

[24] Poullain V, Gandon S, Brockhurst MA, Buckling A, Hochberg ME. 2008 The evolution of specificity in evolving and coevolving antagonistic interactions between a bacteria and its phage. Evolution **62**, 1-11. (10.1111/j.1558-5646.2007.00260.x) 18005153

[25] Goehlich H, Roth O, Wendling CC. 2019 Filamentous phages reduce bacterial growth in low salinities. R. Soc. Open Sci. **6**, 191669. (10.1098/rsos.191669) 31903215 PMC6936277

[26] Harrison E, Guymer D, Spiers AJ, Paterson S, Brockhurst MA. 2015 Parallel compensatory evolution stabilizes plasmids across the parasitism-mutualism continuum. Curr. Biol. **25**, 2034-2039. (10.1016/j.cub.2015.06.024) 26190075

[27] Lenski RE, Rose MR, Simpson SC, Tadler SC. 1991 Long-term experimental evolution in *Escherichia coli*.1. Adaptation and divergence during 2,000 generations. Am. Nat. **138**, 1315-1341. (10.1086/285289)

[28] Cumby N, Edwards AM, Davidson AR, Maxwell KL. 2012 The bacteriophage HK97 gp15 moron element encodes a novel superinfection exclusion protein. J. Bacteriol. **194**, 5012-5019. (10.1128/JB.00843-12) 22797755 PMC3430355

[29] Susskind MM, Wright A, Botstein D. 1974 Superinfection exclusion by P22 prophage in lysogens of *Salmonella typhimurium*. IV. Genetics and physiology of *sie*B exclusion. Virology **62**, 367-384. (10.1016/0042-6822(74)90399-7) 4610993

[30] Uc-Mass A, Loeza EJ, de la Garza M, Guarneros G, Hernandez-Sanchez J, Kameyama L. 2004 An orthologue of the *cor* gene is involved in the exclusion of temperate lambdoid phages. Evidence that Cor inactivates FhuA receptor functions. Virology **329**, 425-433. (10.1016/j.virol.2004.09.005) 15518820

[31] Sun X, Gohler A, Heller KJ, Neve H. 2006 The *ltp* gene of temperate *Streptococcus thermophilus* phage TP-J34 confers superinfection exclusion to *Streptococcus thermophilus* and *Lactococcus lactis*. Virology **350**, 146-157. (10.1016/j.virol.2006.03.001) 16643978

[32] Bull JJ, Vegge CS, Schmerer M, Chaudhry WN, Levin BR. 2014 Phenotypic resistance and the dynamics of bacterial escape from phage control. PLoS ONE **9**, e94690. (10.1371/journal.pone.0094690) 24743264 PMC3990542

[33] Martinez E, Campos-Gomez J. 2016 Pf Filamentous phage requires UvrD for replication in *Pseudomonas aeruginosa*. mSphere **1**, e00104-15. (10.1128/mSphere.00104-15) PMC4863604 27303696

[34] Leon M, Bastias R. 2015 Virulence reduction in bacteriophage resistant bacteria. Front. Microbiol. **6**, 343. (10.3389/fmicb.2015.00343) 25954266 PMC4407575

[35] Proft T, Baker EN. 2009 Pili in gram-negative and gram-positive bacteria — structure, assembly and their role in disease. Cell. Mol. Life Sci. **66**, 613-635. (10.1007/s00018-008-8477-4) 18953686 PMC11131518

[36] Wong A, Rodrigue N, Kassen R. 2012 Genomics of adaptation during experimental evolution of the opportunistic pathogen *Pseudomonas aeruginosa*. PLoS Genet. **8**, e1002928. (10.1371/journal.pgen.1002928) 23028345 PMC3441735

[37] McElroy KE, Hui JG, Woo JK, Luk AW, Webb JS, Kjelleberg S, Rice SA, Thomas T. 2014 Strain-specific parallel evolution drives short-term diversification during *Pseudomonas aeruginosa* biofilm formation. Proc. Natl Acad. Sci. USA **111**, E1419-E1427. (10.1073/pnas.1314340111) 24706926 PMC3986123

[38] Roux S et al. 2019 Cryptic inoviruses revealed as pervasive in bacteria and archaea across Earth's biomes. Nat. Microbiol. **4**, 1895-1906. (10.1038/s41564-019-0510-x) 31332386 PMC6813254

[39] Lerner TJ, Model P. 1981 The ‘steady state’ of coliphage f1: DNA synthesis late in infection. Virology **115**, 282-294. (10.1016/0042-6822(81)90111-2) 7032054

[40] Waldor MK, Mekalanos JJ. 1996 Lysogenic conversion by a filamentous phage encoding cholera toxin. Science **272**, 1910-1914. (10.1126/science.272.5270.1910) 8658163

[41] Gonzalez MD, Lichtensteiger CA, Caughlan R, Vimr ER. 2002 Conserved filamentous prophage in *Escherichia coli* O18:K1:H7 and *Yersinia pestis* biovar orientalis. J. Bacteriol. **184**, 6050-6055. (10.1128/JB.184.21.6050-6055.2002) 12374839 PMC135385

[42] Rice SA et al. 2009 The biofilm life cycle and virulence of *Pseudomonas aeruginosa* are dependent on a filamentous prophage. ISME J. **3**, 271-282. (10.1038/ismej.2008.109) 19005496 PMC2648530

[43] Secor PR et al. 2015 Filamentous bacteriophage promote biofilm assembly and function. Cell Host Microbe **18**, 549-559. (10.1016/j.chom.2015.10.013) 26567508 PMC4653043

[44] Sweere JM et al. 2019 Bacteriophage trigger antiviral immunity and prevent clearance of bacterial infection. Science **363**, 1416. (10.1126/science.aat9691) PMC6656896 30923196

[45] Wendling CC, Refardt D, Hall AR. 2020 Fitness benefits to bacteria of carrying prophages and prophage-encoded antibiotic-resistance genes peak in different environments. Evolution **75**, 515-528. (10.1111/evo.14153) PMC7986917 33347602

[46] Chouikha I, Charrier L, Filali S, Derbise A, Carniel E. 2010 Insights into the infective properties of Ypf Φ, the *Yersinia pestis* filamentous phage. Virology **407**, 43-52. (10.1016/j.virol.2010.07.048) 20728914

[47] Derbise A, Chenal-Francisque V, Pouillot F, Fayolle C, Prevost MC, Medigue C, Hinnebusch BJ, Carniel E. 2007 A horizontally acquired filamentous phage contributes to the pathogenicity of the plague bacillus. Mol. Microbiol. **63**, 1145-1157. (10.1111/j.1365-2958.2006.05570.x) 17238929

[48] Bobay LM, Touchon M, Rocha EPC. 2014 Pervasive domestication of defective prophages by bacteria. Proc. Natl Acad. Sci. USA **111**, 12 127-12 132. (10.1073/pnas.1405336111) PMC4143005 25092302

[49] Davis BM, Moyer KE, Boyd EF, Waldor MK. 2000 CTX prophages in classical biotype *Vibrio cholerae*: functional phage genes but dysfunctional phage genomes. J. Bacteriol. **182**, 6992-6998. (10.1128/JB.182.24.6992-6998.2000) 11092860 PMC94825

[50] Wendling CC et al. 2022 Data from: Higher phage virulence accelerates the evolution of host resistance. Dryad Digital Repository. (10.5061/dryad.kprr4xh6c) PMC9532999 36196537

[51] Wendling CC et al. 2022 Data from: Higher phage virulence accelerates the evolution of host resistance. Figshare. (10.6084/m9.figshare.c.6198513) PMC9532999 36196537

